# Supplementary material for: Screening of Palladium/Charcoal Catalysts for Hydrogenation of Diene Carboxylates with Isolated-Rings (Hetero)aliphatic Scaffold
Source: Molecules. 2023 Jan 26;28(3):1201. doi: 10.3390/molecules28031201 (PMC9920177; doi:10.3390/molecules28031201)
Supplement: Supplementary file 1 [file molecules-28-01201-s001.zip › molecules-2102012-supplementary.pdf]

Supporting Information

Screening of Palladium/Charcoal Catalysts for Hydrogenation of Diene Carboxylates with Isolated-Rings (Hetero)aliphatic Scaffold

Vladyslav V. Subbotin[1,2], Bohdan V. Vashchenko[1,3], Vitalii M. Asaula[2], Eduard V. Verner[1,2], Mykyta O. Ivanytsya[1,2], Olexiy Shvets[2], Eugeniy N. Ostapchuk[1,3], Oleksandr O. Grygorenko[1,3], Sergey V. Ryabukhin[1,3,4], Dmitriy M. Volochnyuk[1,3,4], Sergey V. Kolotilov*[1,2,3]

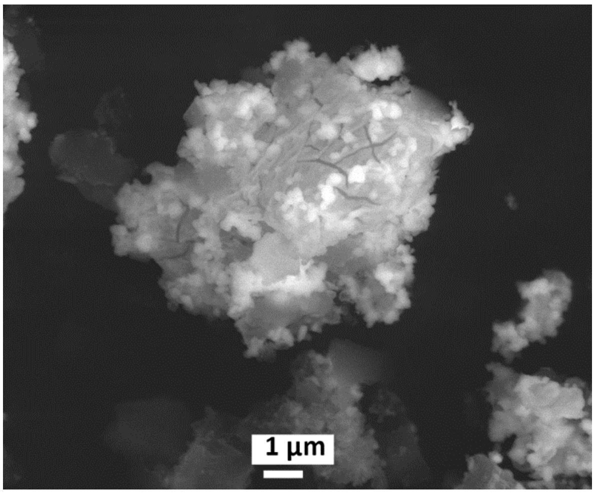

Figure S1. SEM image in backscattered electron mode for $Pd(OH)_2/C$-2 showing one particle covered by hydrated PdO.

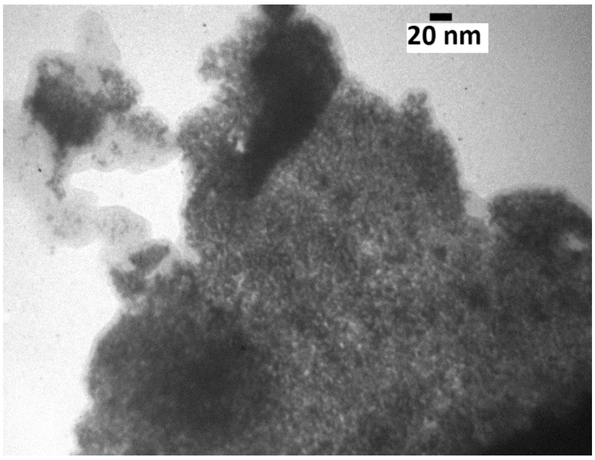

Figure S2. TEM image of $Pd(OH)_2/C$-2 after hydrogenation reaction.

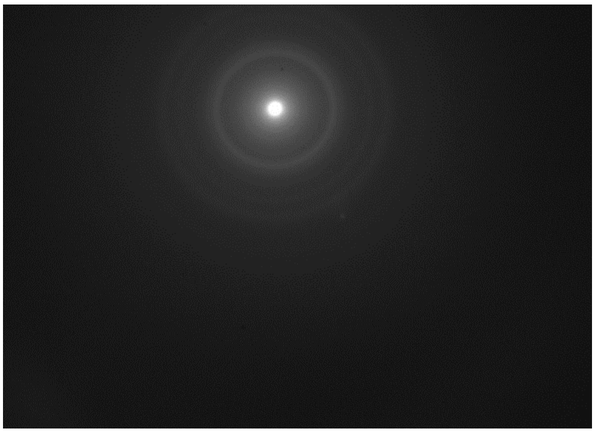

Figure S3. Electron diffraction pattern of $Pd(OH)_2$/C-2 after hydrogenation reaction.

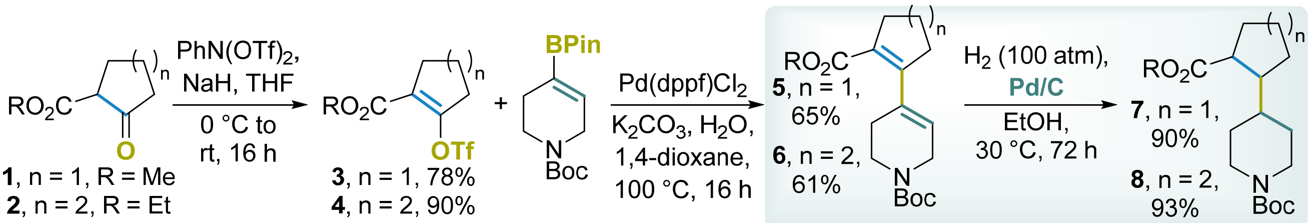

Figure S4. Reaction scheme for preparation of **1** and **2**

The general procedure for the preparation of alkenyl triflates. 0.2 M solution of the corresponding α-ketoester (57.0 mmol) in THF (285) was cooled to 0 °C, and NaH (60% in mineral oil, 2.30 g) was added in portions. The reaction mixture was stirred at 0 °C for 30 min, then $PhN(OTf)_2$ (20.5 g) was added. The resulting mixture stirred at rt overnight, then poured into H_2O (500 mL) and extracted with EtOAc (2 × 300 mL). Combined organic layers were washed with brine (2 × 100 mL), dried over Na_2SO_4, filtered, and evaporated in *vacuo* to dryness. The crude product was purified via column chromatography on silica gel using hexanes – EtOAc (20:1, v/v) as an eluent.

The general procedure for the Suzuki cross-coupling reaction. The corresponding triflate (3.65 mmol) was dissolved in 1,4-dioxane – H_2O (14 mL, 7:2, v/v), then K_2CO_3 (1.51 g, 10.9 mmol), $Pd(dppf)Cl_2$ (267 mg, 0.365 mmol), and *tert*-butyl 4-(4,4,5,5-tetramethyl-1,3,2-dioxaborolan-2-yl)-3,6-dihydropyridine-1(2*H*)-carboxylate (1.24 g, 4.01 mmol). The reactor was degassed, purged with Ar, and the reaction mixture was stirred at 100 °C for 18 h. The resulting mixture was cooled to rt, EtOAc (40 mL) was added, the resulting solution was washed with H_2O (3 × 15 mL) and brine (3 × 15 mL), dried over Na_2SO_4, filtered, and evaporated in *vacuo* to dryness. The crude product was purified via column chromatography on silica gel using hexanes – EtOAc (20:1, v/v) as an eluent.

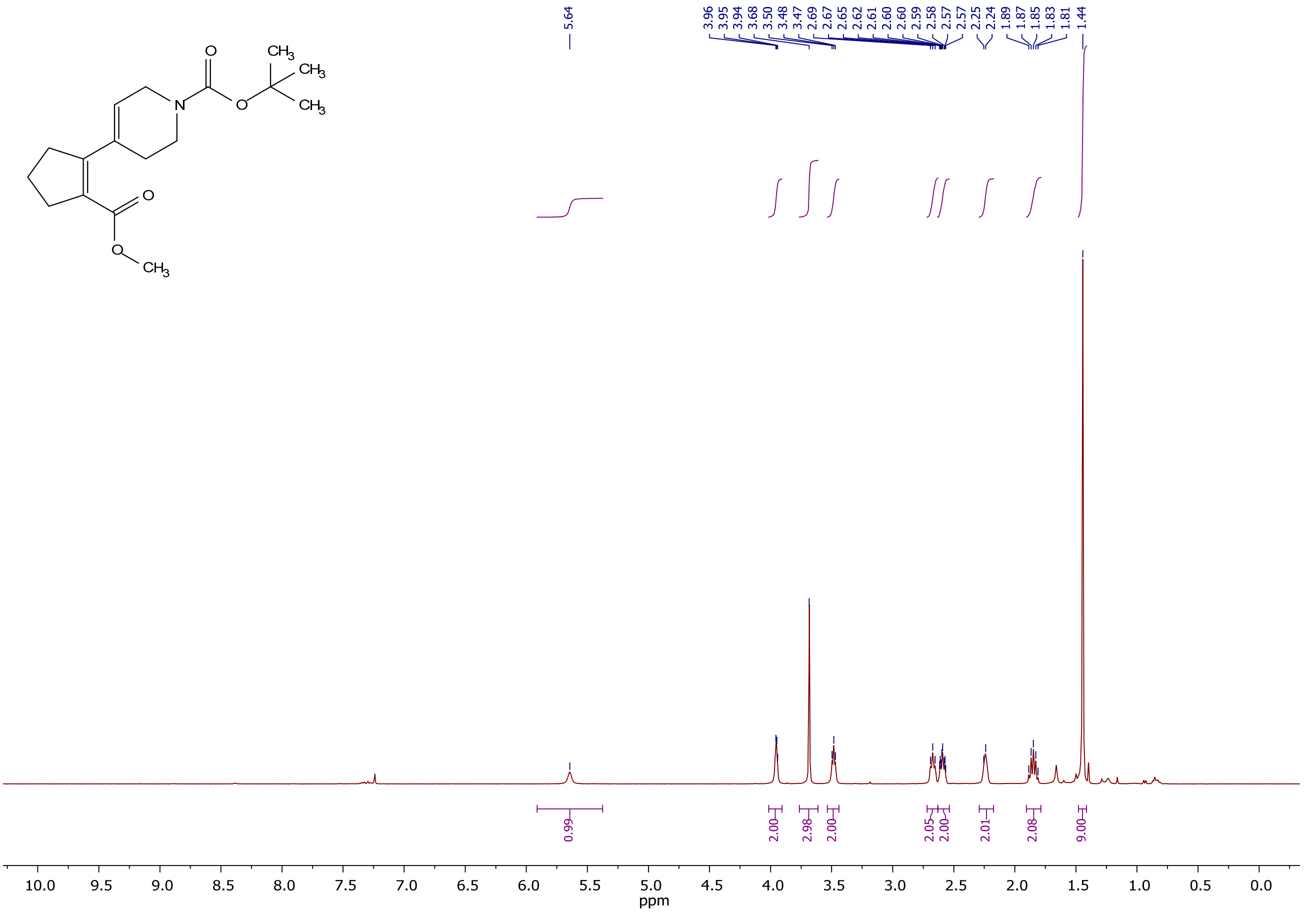

Figure S5. ^{1}H NMR of *tert*-Butyl 4-(2-(methoxycarbonyl)cyclopent-1-en-1-yl)-5,6-dihydropyridine-1(2*H*)-carboxylate (**1**) (400 MHz, $CDCl_3$)

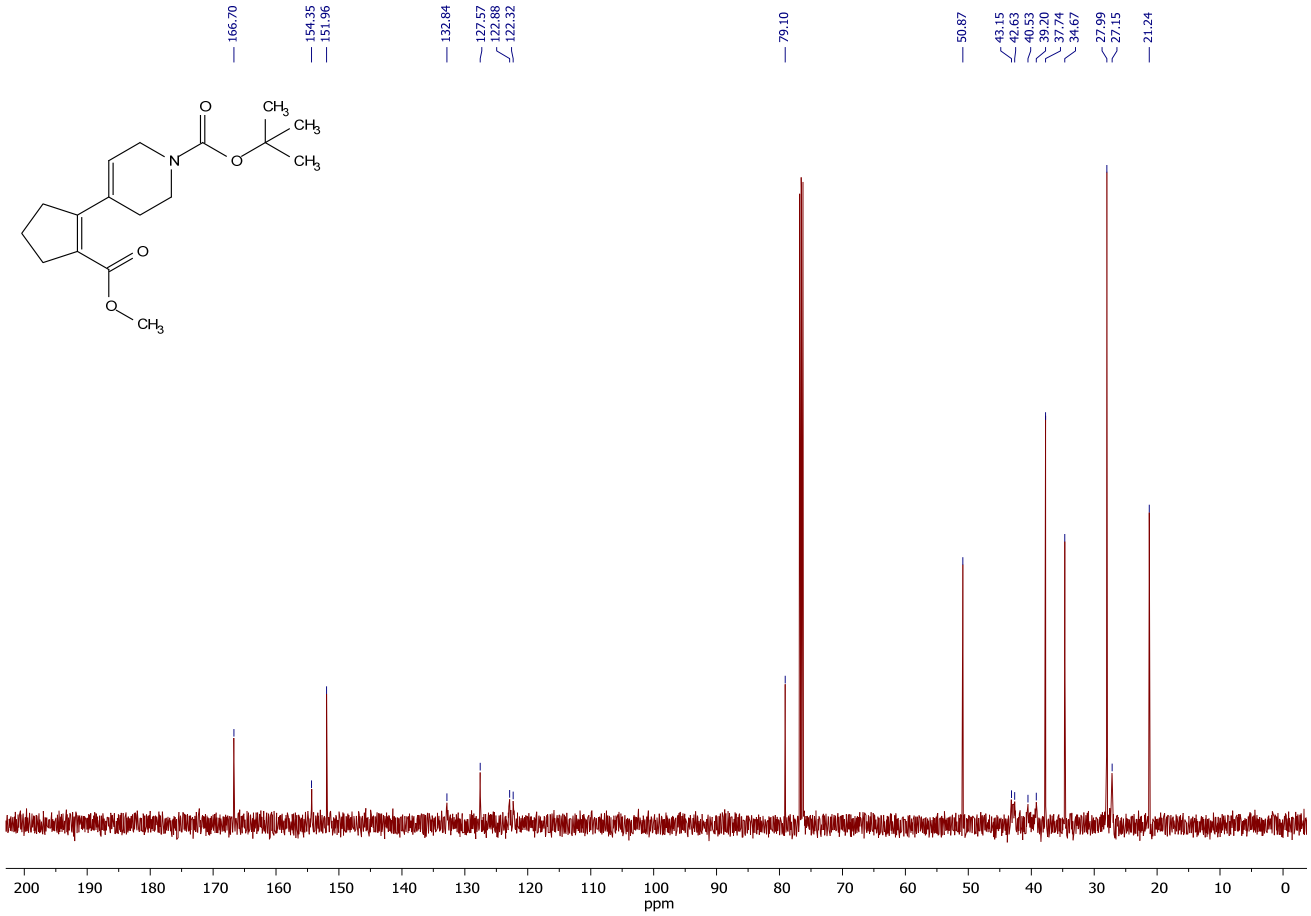

Figure S6. ^{13}C NMR of *tert*-Butyl 4-(2-(methoxycarbonyl)cyclopent-1-en-1-yl)-5,6-dihydropyridine-1(2*H*)-carboxylate (**1**) (126 MHz, $CDCl_3$)

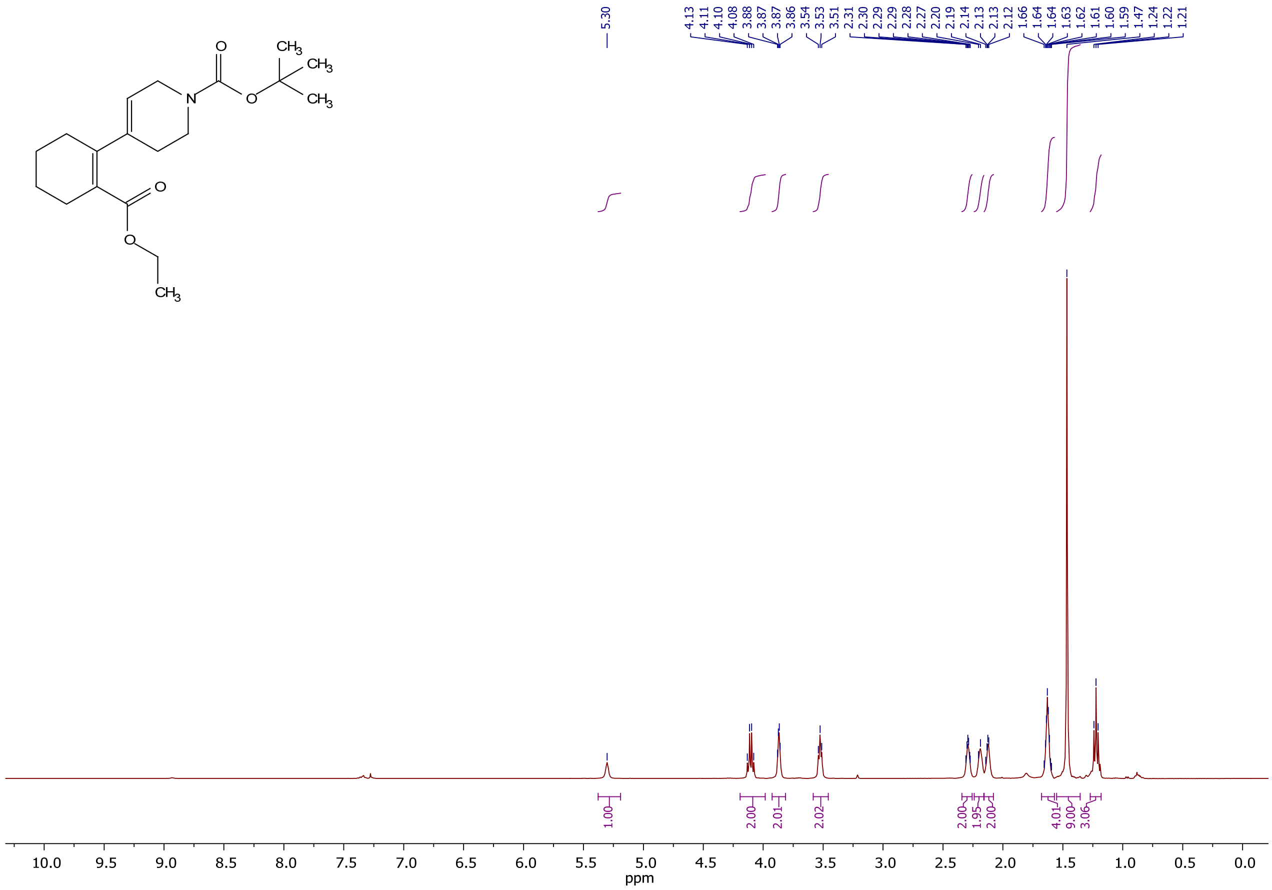

Figure S7. ^{1}H NMR of *tert*-Butyl 4-(2-(ethoxycarbonyl)cyclohex-1-en-1-yl)-5,6-dihydropyridine-1(2*H*)-carboxylate (**2**) (400 MHz, $CDCl_3$)

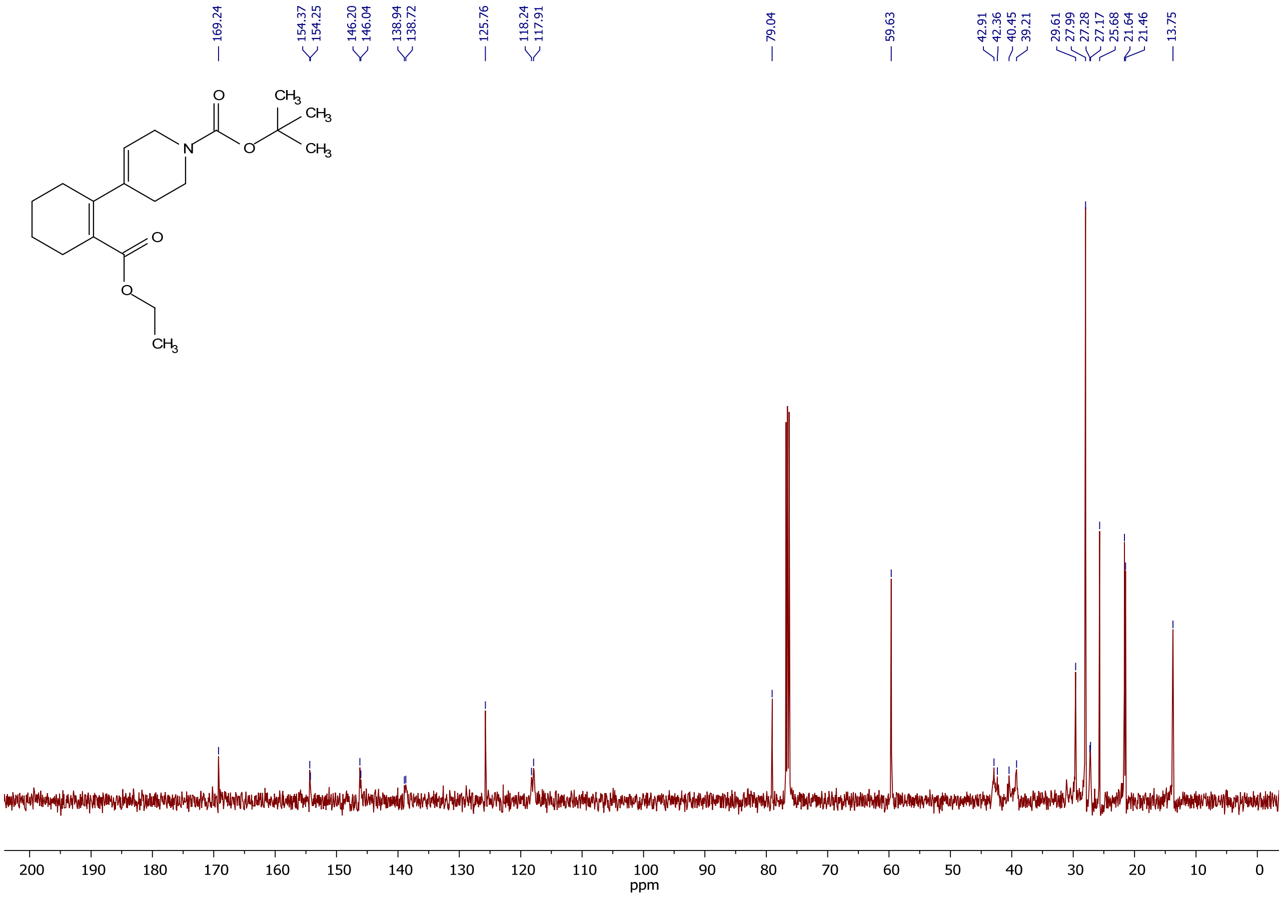

Figure S8. ^{13}C NMR of *tert*-Butyl 4-(2-(ethoxycarbonyl)cyclohex-1-en-1-yl)-5,6-dihydropyridine-1(2*H*)-carboxylate (2) (126 MHz, $CDCl_3$)

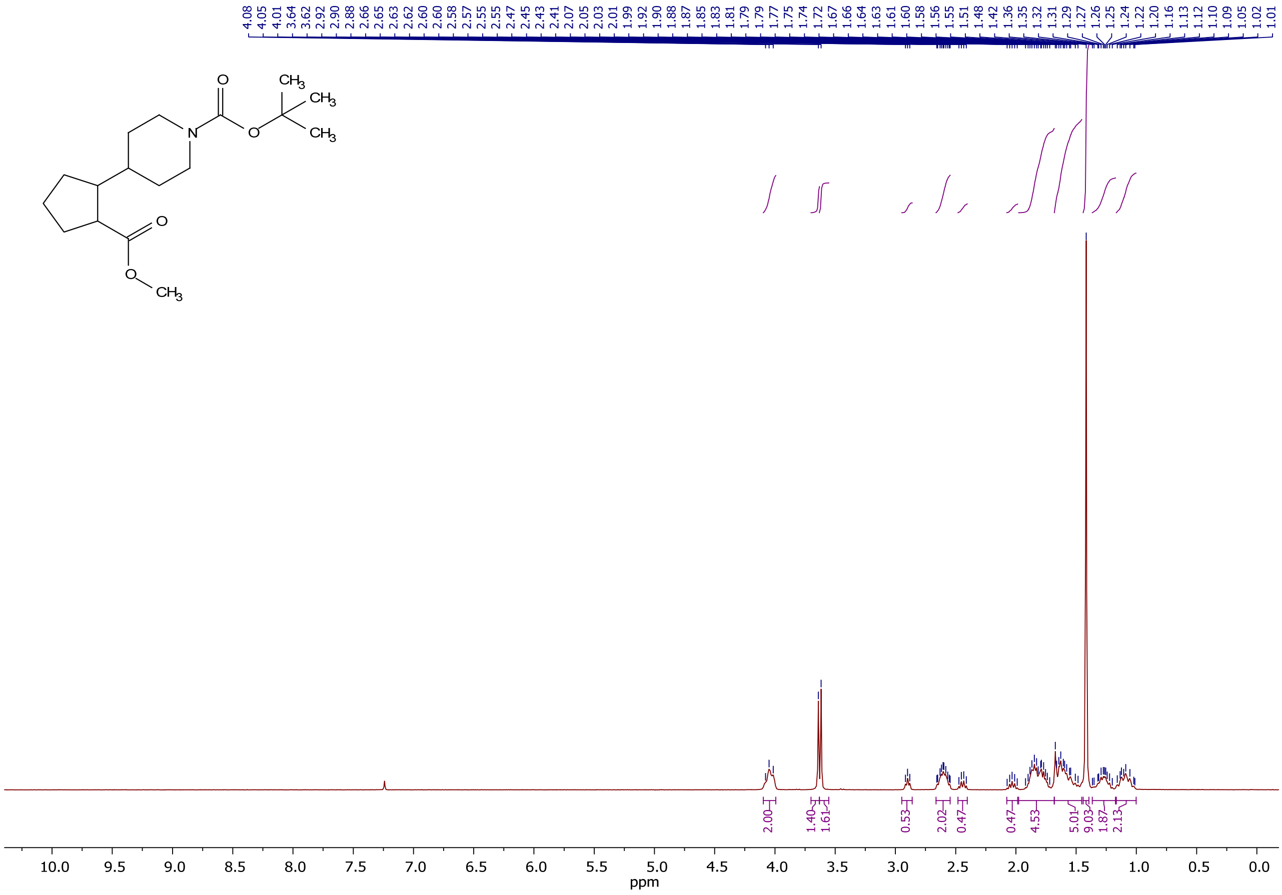

Figure S9. ^{1}H NMR of *tert*-Butyl 4-(2-(ethoxycarbonyl)cyclopentyl)piperidine-1-carboxylate (3, ca. 8:7 mixture of diastereomers) (400 MHz, $CDCl_3$)

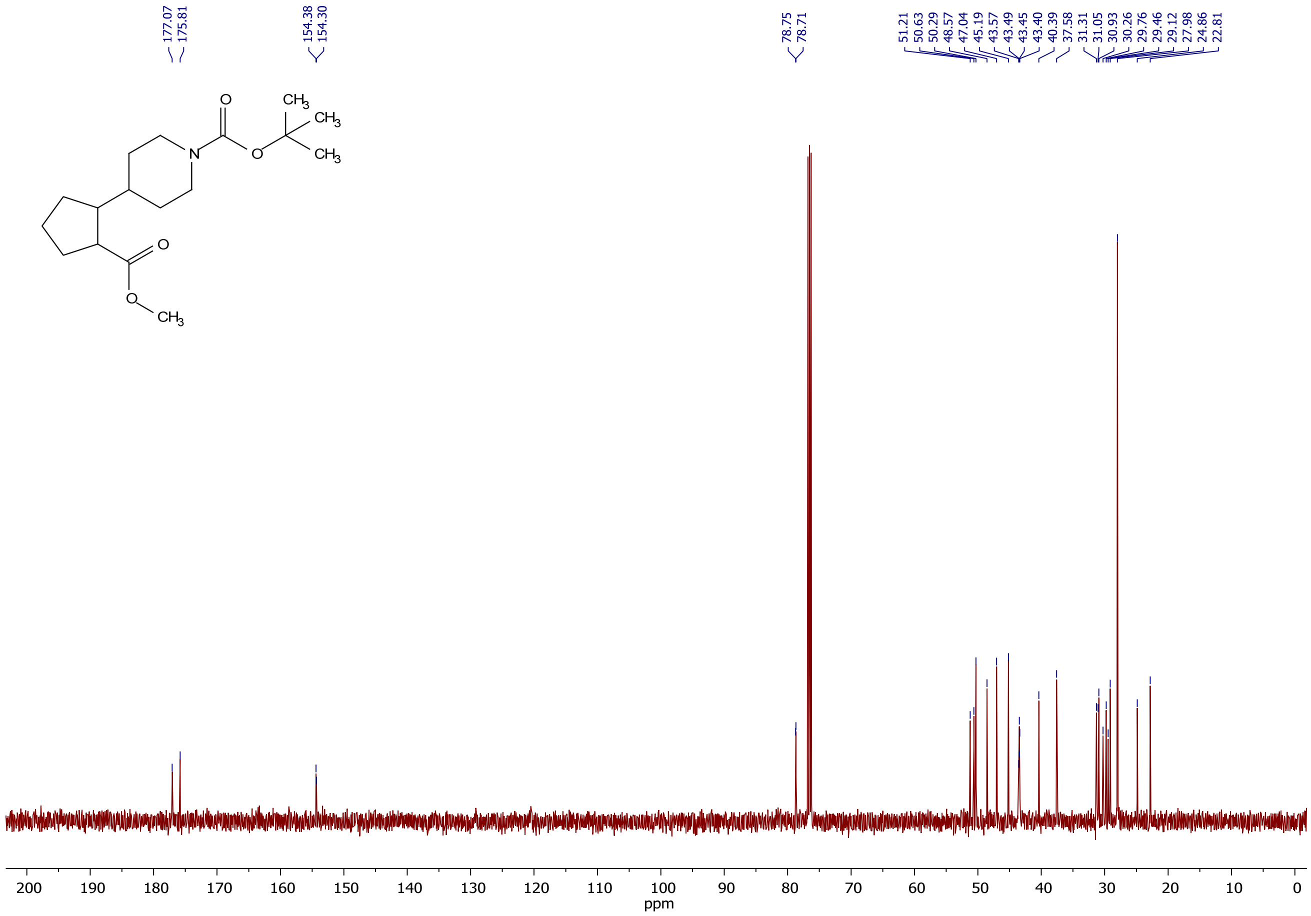

Figure S10. ^{13}C NMR of *tert*-Butyl 4-(2-(ethoxycarbonyl)cyclopentyl)piperidine-1-carboxylate (3, ca. 8:7 mixture of diastereomers) (126 MHz, $CDCl_3$)

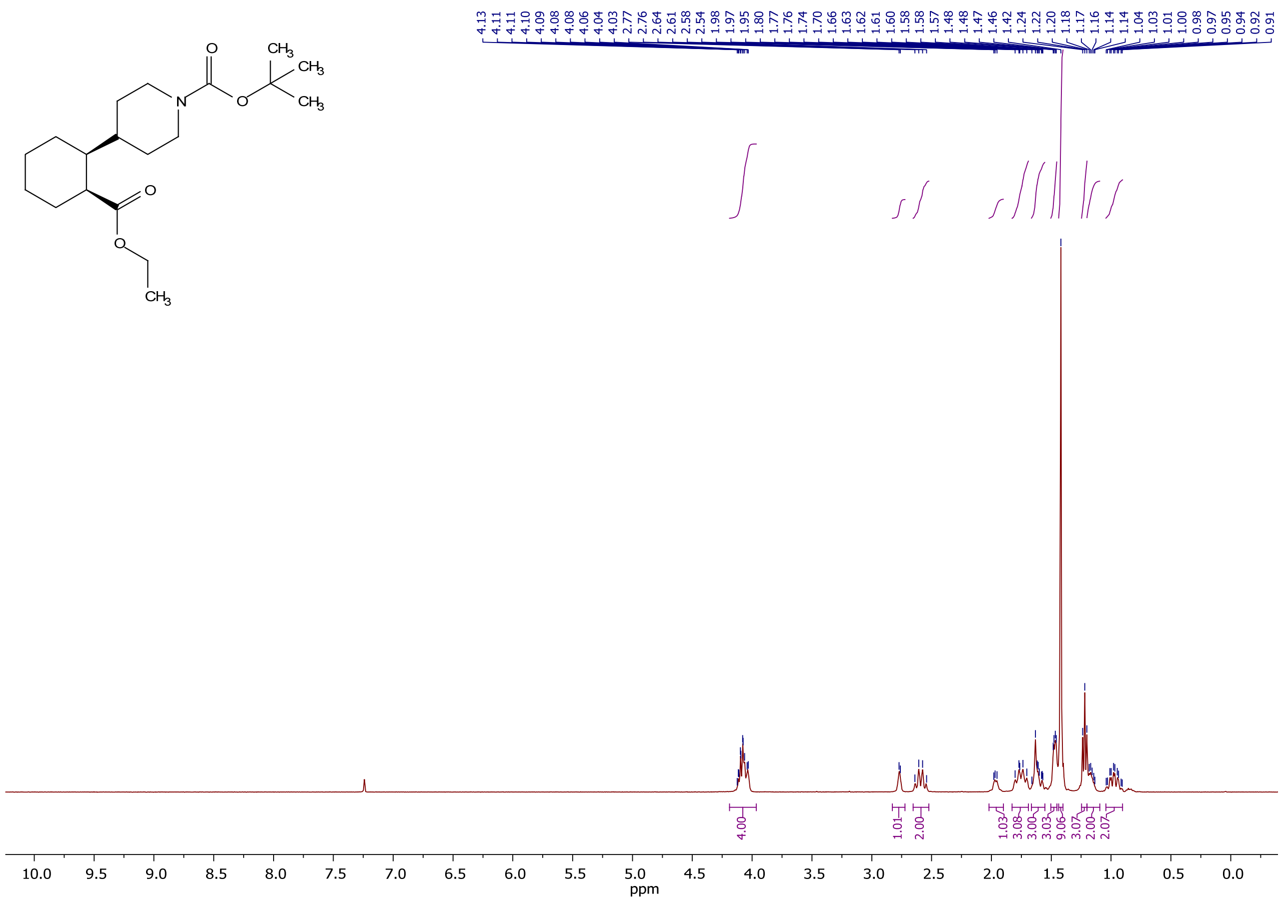

Figure S11. ^{1}H NMR of *cis-tert*-Butyl 4-(2-(ethoxycarbonyl)cyclohexyl)piperidine-1-carboxylate (**4**) (400 MHz, $CDCl_3$)

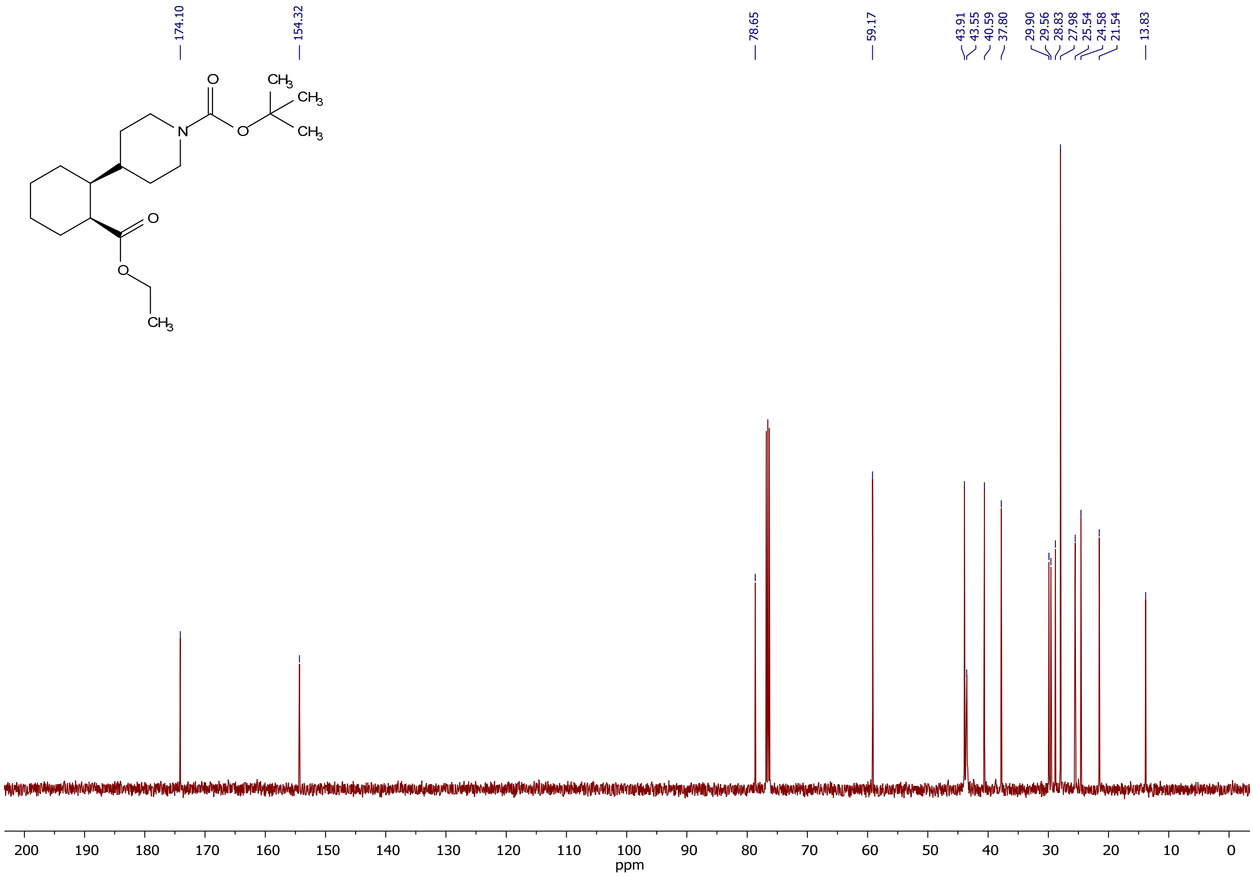

Figure S12. ^{13}C NMR of *cis-tert*-Butyl 4-(2-(ethoxycarbonyl)cyclohexyl)piperidine-1-carboxylate (**4**) (126 MHz, $CDCl_3$)

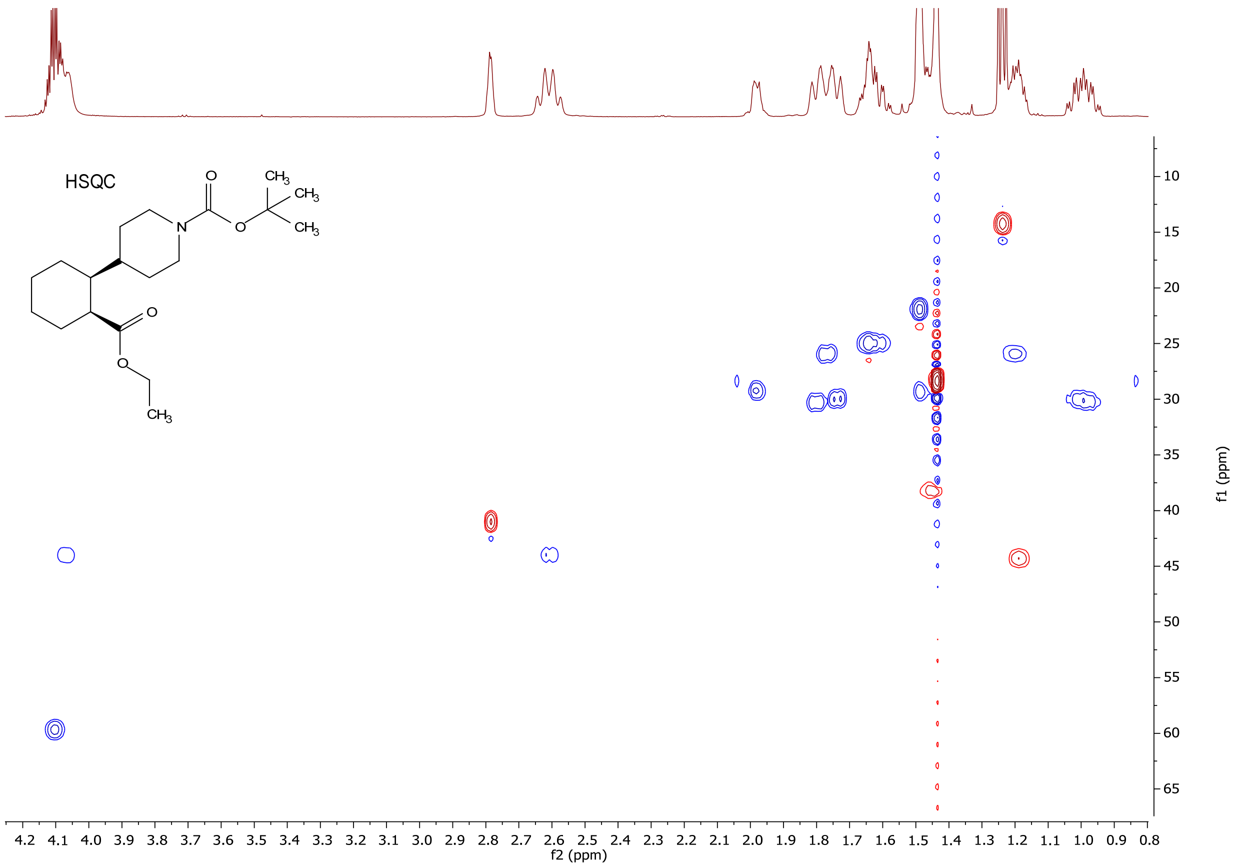

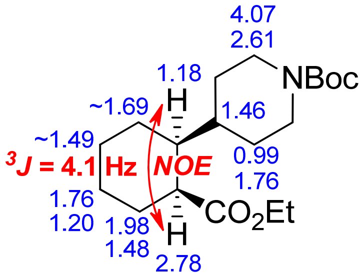

Figure S13. HSQC of *cis-tert*-Butyl 4-(2-(ethoxycarbonyl)cyclohexyl)piperidine-1-carboxylate (**4**)

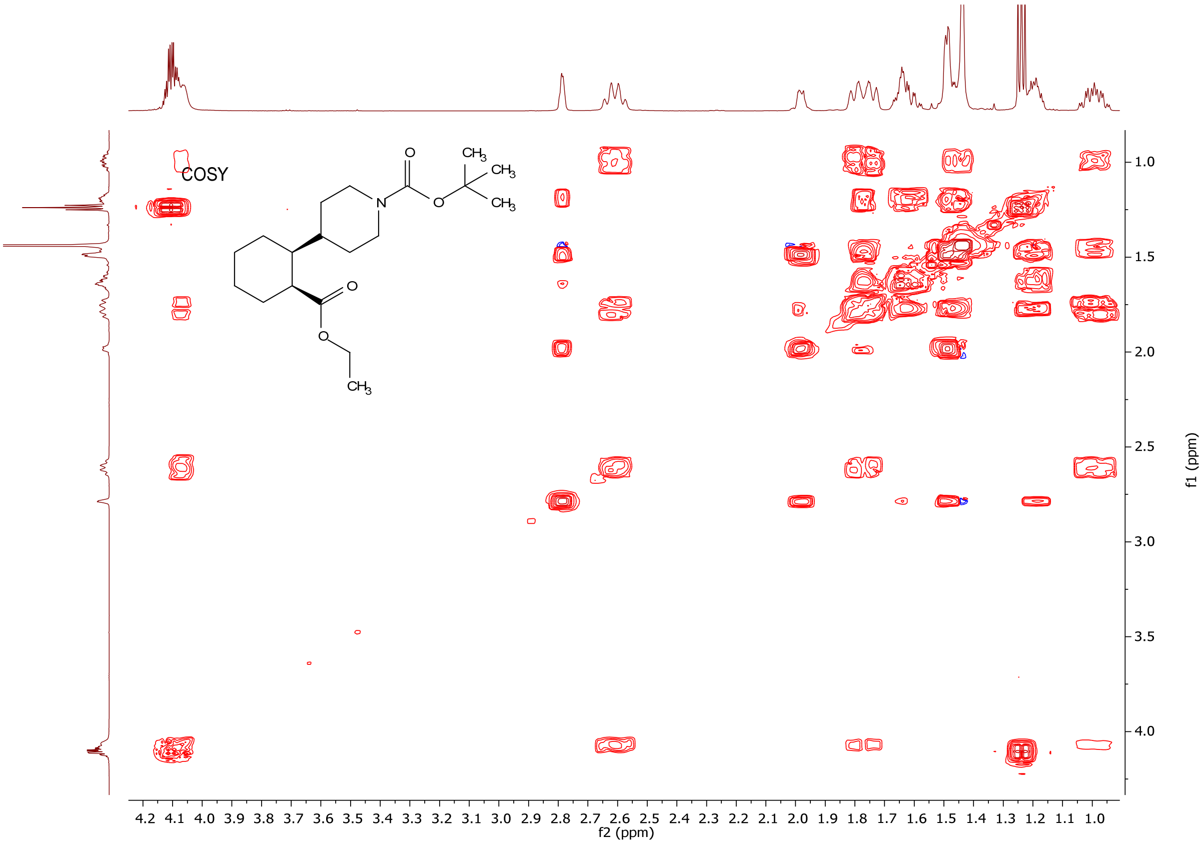

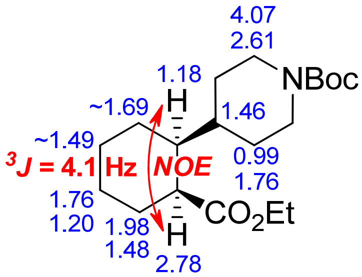

Figure S14. COSY of *cis-tert*-Butyl 4-(2-(ethoxycarbonyl)cyclohexyl)piperidine-1-carboxylate (**4**)

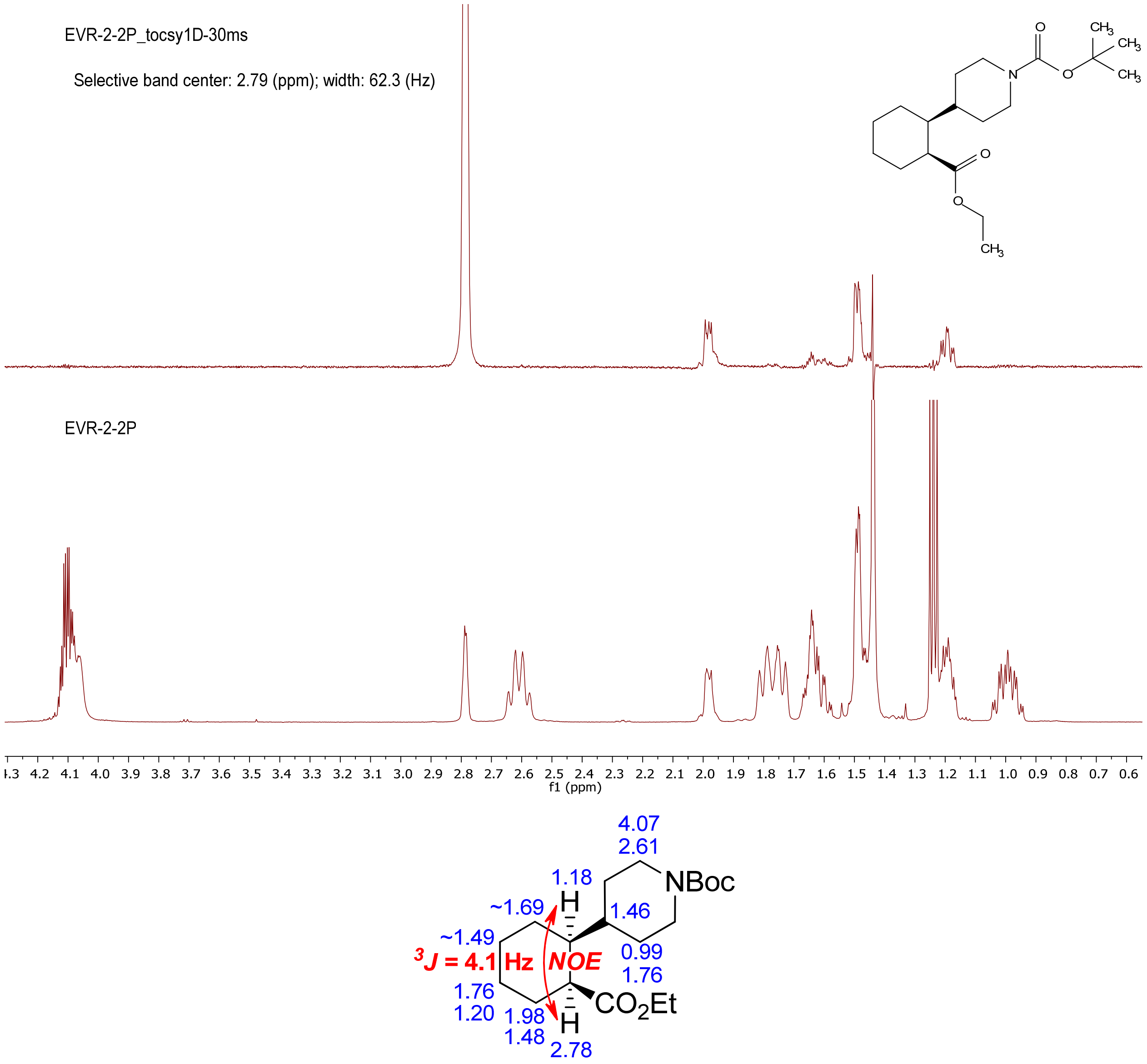

Figure S15. TOCSY of *cis*-*tert*-Butyl 4-(2-(ethoxycarbonyl)cyclohexyl)piperidine-1-carboxylate (**4**)

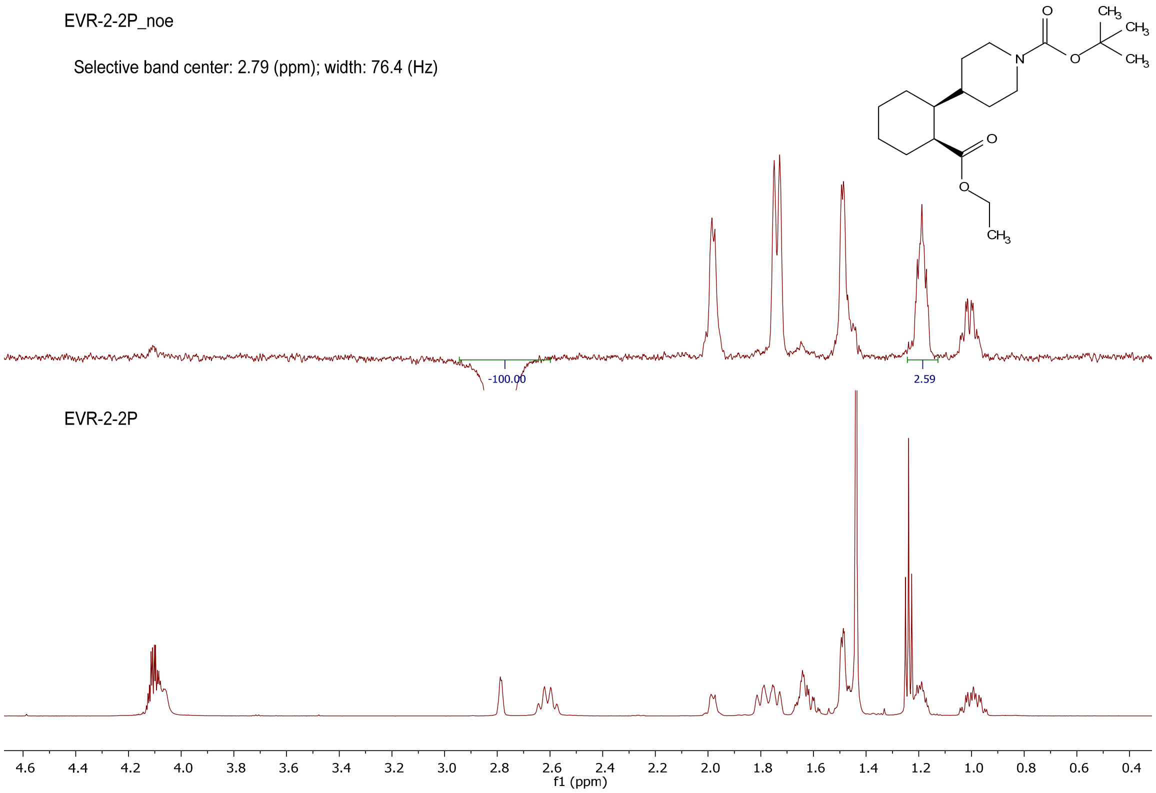

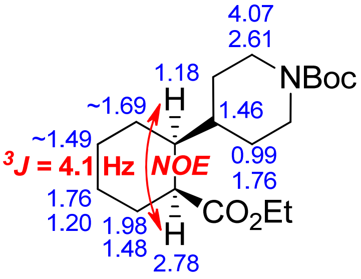

Figure S16. H–H NOE of *cis*-*tert*-Butyl 4-(2-(ethoxycarbonyl)cyclohexyl)piperidine-1-carboxylate (**4**)

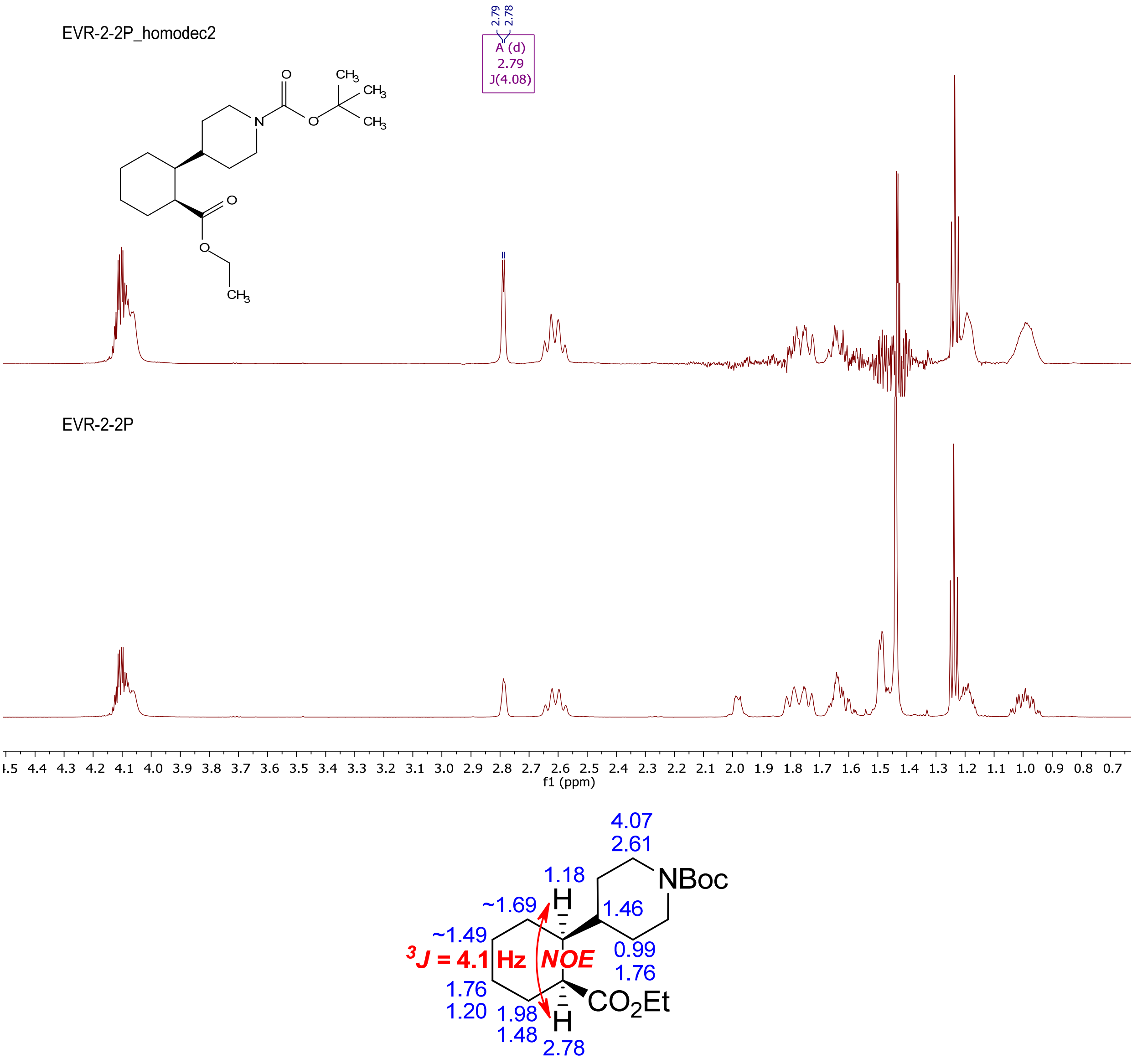

Figure S17. Homodecoupling spectra of *cis*-*tert*-Butyl 4-(2-(ethoxycarbonyl)cyclohexyl)piperidine-1-carboxylate (**4**)
